# Navigating Adolescence with a Chronic Health Condition: A Perspective on the Psychological Effects of HAIR-AN Syndrome on Adolescent Girls

**DOI:** 10.1100/tsw.2006.242

**Published:** 2006-10-23

**Authors:** Kimberly K. McClanahan, Hatim A. Omar

**Affiliations:** Section of Adolescent Medicine, Department of Pediatrics, University of Kentucky, Lexington, KY, USA

**Keywords:** HAIR-AN syndrome, female adolescent development, mental health, adolescence, mental health issues, chronic health conditions

## Abstract

HAIR-AN syndrome is a subphenotype of polycystic ovary syndrome and is characterized by acne, obesity, hirsutism, and acanthosis nigricans. It usually manifests in early adolescence, a time of significant developmental change in females across physical, cognitive, social, and emotional domains. We contend that adolescent development for females is difficult, even in the best of circumstances, and having a chronic health condition, like HAIR-AN syndrome, will likely impact the afflicted individuals development and psychological well-being. While many researchers have discussed the long-term health effects of HAIR-AN and similar disorders, little has been written about the potential psychological sequelae of HAIR-AN on the adolescent girl. We discuss the normal developmental sequence for adolescent girls across early, middle, and late adolescence; discuss common mental health problems that adolescents experience; define HAIR-AN syndrome and its clinical manifestations; and discuss its likely psychological impact on adolescent girls. We also make suggestions for future clinical interventions and research in the area of HAIR-AN syndrome and its psychological sequelae.

### INTRODUCTION

Adolescence is a time that is defined most simply by the word "change". Not since infancy has there been such rapid growth and development. Changes occur across physical, cognitive, social, and emotional domains, and each stage of adolescence (i.e., early, middle, late) brings with it unique developmental goals and potential interruption or suspension of those goals toward adulthood. Disruptions in development can be influenced by many things, including physical and/or medical problems[1], and those problems can have a significant impact on healthy psychological development.

## ADOLESCENT DEVELOPMENT IN FEMALES

### Developmental Tasks of Early Adolescence

Early adolescence is characterized by major physical changes with the onset of puberty, maturation of sexual organs and development of secondary sex characteristics, and the onset of menstruation in females. Cognitive changes occur as young adolescents begin the transition from concrete to formal operational thought (i.e., abstract thinking)[2,3,4], including a limited ability to think hypothetically and to take multiple perspectives[5,6,7]. Until early adolescence, children think concretely, utilizing a present orientation and a tendency to view choices as black or white, with little appreciation of shades of gray. Concrete thinkers' ability to project into the future is limited, and they are often unable to perceive the long-range implications of current decisions. This means that although they may have the cognitive information they need to make decisions, they may have difficulty applying that information to their particular situation[6]. As children reach early adolescence, they begin to think abstractly at times and can have an orientation toward the future, perceive implications of actions, and look at things from the perspective of others. However, these capacities fluctuate and do not consolidate fully until late adolescence.

Early adolescence also elicits changes in the social and emotional domains. Social roles are largely defined by external sources, and social relationships are centered in the peer group with group values tending to govern individual behavior. Acceptance by peers is critical to self-esteem. Some early adolescents will begin to experiment with sexual behavior, but many early adolescents are not sexually active with other youth[6]. The emotional domain finds the early adolescent strongly identified with a peer group and dependent on it for emotional stability, support, and molding of emerging identity. Self-esteem is greatly affected by the acceptance of peers. Hormonal changes tend to increase early adolescents' emotional lability, and they may show exaggerated affect and frequent mood swings.

### Developmental Tasks of Middle and Late Adolescence

Middle and late adolescence bring continued developmental changes. In middle adolescence, physical maturation continues and cognitive development progresses with advancement toward full formal operational thinking, thus allowing a teen to think abstractly more consistently. By the time an adolescent reaches late adolescence, most physical and cognitive changes have occurred, but social and emotional changes continue. As a teen reaches middle and late adolescence, social values are more likely to become individualized and internalized, after careful consideration and independent thought. Friends are more often selected on personal characteristics and mutual interests rather than some external criterion (e.g., popularity). Having a large peer group declines in importance, individual friendships are strengthened, and more youth "date" in one-on-one relationships[6]. The emotional domain finds the older adolescent with a more individualized identity, a sense of self that is separate from family and peer group, and self-esteem more influenced by the ability to live up to internalized standards for behavior rather than acceptance or rejection by others. Self-assessment and introspection are common and full operational thought will likely be achieved by the end of late adolescence, thus allowing the young person to consistently have future orientation, view the implications of decisions and circumstances, and place oneself in someone else's position. Table 1 shows a summary of normative changes throughout adolescence.

## CHALLENGES TO OPTIMAL ADOLESCENT FEMALE DEVELOPMENT

The health status of adolescents has recently become a concern for researchers, clinicians, and policy makers[8], and the concern is greater for adolescent girls than for boys because "Girls today are much more

**TABLE 1**
**A Summary of Normative Changes Throughout Adolescence.**

| Early Adolescence (11–14 Years) | Middle Adolescence (14–17 Years) | Late Adolescence (17–20 Years) |
|---|---|---|
| PHYSICAL | | |
| Rapidly accelerating growth<br>Reaches peak velocity<br>Secondary sex characteristics appear | Growth decelerating in girls<br>Stature reaches 95% of adult height<br>Secondary sex characteristics well advanced | Physically mature<br>Structure and reproductive growth almost complete |
| COGNITIVE | | |
| Explores newfound ability for limited abstract thought<br>Struggling to define new values<br>Comparison of "normality" with peers of the same sex | Developing capacity for abstract thinking<br>Enjoys intellectual powers, often in idealistic terms<br>Concern with philosophical, political, and social problems | Established abstract thought<br>Can perceive and act on long-range goals<br>Able to view problems comprehensively<br>Intellectual and functional identity established |
| SOCIAL: RELATIONSHIPS WITH PEERS | | |
| Seeks peer affiliations to counter instability generated by rapid change<br>Upsurge of close, idealized friendships with members of peer group<br>Struggle for mastery takes place within peer group | Strong need for identity to affirm self-image<br>Behavioral standards set by peer group<br>Acceptance by peers extremely important; fear of rejection<br>Exploration of ability to attract others (individuals) | Peer group recedes in importance in favor of individual friendships<br>Testing of intimate relationships against possibility of permanent alliance<br>Relationships characterized by giving and sharing |
| SOCIAL: SEXUALITY | | |
| Self-exploration and evaluation<br>Limited dating, usually group<br>Limited intimacy | Multiple plural relationships<br>Decisive turn toward heterosexuality or homosexuality<br>Exploration of "self-appeal"<br>Feeling of being "in love"<br>Tentative establishment of relationships | Forms stable relationships and attachment to another<br>Growing capacity for mutuality and reciprocity<br>Dating as pair<br>Intimacy involves commitment rather than exploration and romanticism |
| EMOTIONAL: IDENTITY | | |
| Preoccupied with rapid body changes<br>Trying out various roles<br>Measurement of attractiveness by acceptance or rejection of peers<br>Conformity to group norms | Modifies body image<br>Very self-centered, increased narcissism<br>Tendency toward inner experience and self-discovery<br>Has rich fantasy life<br>Idealistic<br>Able to perceive future implications of current behavior and decisions; variable application | Body image and gender role definition nearly secured<br>Mature sexual identity<br>Phase of consolidation of identity<br>Stability of self-esteem<br>Comfortable with physical growth<br>Social roles defined and articulated |
| EMOTIONAL: RELATIONSHIP WITH PARENTS | | |
| Defining independence/dependence boundaries<br>Strong desire to remain dependent on parents while trying to detach<br>No major conflicts over parental control | Major conflicts over independence and control<br>Low point in parent/child relationship<br>Greatest push for emancipation; disengagement<br>Final and irreversible emotional detachment from parents; mourning | Emotional and physical separation from parents completed<br>Independence from family with less conflict<br>Emancipation nearly secured |
| EMOTIONAL: PSYCHOLOGICAL HEALTH | | |
| Wide mood swings<br>Intense daydreaming<br>Anger outwardly expressed with moodiness, temper outbursts, and verbal insults | Tendency toward inner experiences; more introspective<br>Tendency to withdraw when upset or feelings are hurt<br>Vacillation of emotions in time and range<br>Feelings of inadequacy common; difficulty in asking for help | More constancy of emotion<br>Anger more apt to be concealed |

Adapted from Monasterio[6].

oppressed. They are coming of age in a more dangerous, sexualized, and media-saturated culture. They face incredible pressures to be beautiful and sophisticated….. As they navigate a more dangerous world, girls are less protected[9]". Girls are expected to be thin, beautiful, flawless, sexy, cookie-cutter images of the super models and actresses they attempt to emulate. The adolescent girl who cannot meet those media-generated standards of perfection may be dismissed by her peer group (and sometimes her family), thus impacting self-esteem, identity, and self-image negatively. Many things impact whether an adolescent girl will meet or approximate the stereotypical expectations of adolescence, including the timing of physical maturation, mental health issues, and chronic health conditions.

### Earlier Physical Maturation

While adolescence is a critical time of development across all four domains, the development associated with the physical domain appears to be occurring at earlier ages, especially for females. In the U.S., 150 years ago, the average age of menarche was 16 years[10]; today, menstruation has been documented to start earlier with mean ages of menarche at 12.06 for non-Hispanic Black girls, 12.25 for Mexican-American girls, and 12.55 for non-Hispanic White girls[11,12]. The onset of puberty has also been shown[6,13] to commence earlier, with 15% of White females and 48% of Black females showing Tanner Stage 2 breast and pubic hair development at age 8 years, representing a significant increase in the number of children entering puberty at earlier ages than previously documented[11,13].

Given the trend of earlier onset of puberty and menarche, an adolescent's physical maturity may or may not be mirrored by cognitive, social, and emotional maturity because each area, while related, is separate from all other areas, and advanced development in one area does not mean equal advancement in all areas. Additionally, age is a clue to, but does not determine, the developmental stage a teenager has reached. In essence, "Although the human organism is reproductively mature in early adolescence, the brain does not reach a fully adult state of development until the end of the teenage years; social maturity lags well behind[10]". Adolescent girls are now more often physically mature while their cognitive, social, and emotional skills and resources continue to develop at a similar pace as they have historically.

### Mental Health Issues

In the realm of emotional and psychological development, today's youth often face challenges in mental health and wellness as they progress from childhood to adolescence to adulthood. Over the past 20 years, the proportion of pediatric patient visits in general pediatric practices with psychosocial problems has increased from 7 to 19%[14]. According to the 1999 U.S. Surgeon General's report on children's mental health, 13% of children and adolescents have anxiety disorders, 6.2% have mood disorders, 10.3% have disruptive disorders, and 2% have substance use disorders, for a total of 20.9% having one or more mental health or substance abuse disorder[1]. Of those needing active mental health interventions, 11% were found to have significant functional impairment and 5% were found to have extreme functional impairment[1]. Other research has found that 27% of children 9, 11, and 13 years of age have mental impairment, and 20% have a diagnosable mental health condition[15]. Further, suicide is the third leading cause of death in the U.S. by children ages 15–24 and suicide attempts reach a peak during the midadolescent years[1,16,17], highlighting the psychological vulnerability adolescents experience. Finally, many children and adolescents present with both physical and mental disorders, and it has been found that the majority of children and adolescents with medical problems have higher levels of mental disorders[18], suggesting that having a chronic health condition may increase the likelihood of mental health issues and concerns.

### HAIR-AN SYNDROME

Clearly, traversing the road from adolescence to adulthood is often a treacherous journey, even in the best of circumstances. Young women must successfully accomplish appropriate development in physical, cognitive, social, and emotional domains, and in so doing, they must determine who they are with regard to values, beliefs, and morals (identity) in contrast to family and friends, and differentiate (individuate) and separate from their families of origin (autonomy). Additionally, they must establish a capacity for intimacy, become comfortable with their sexuality, and accomplish a sense of achievement[19,20]. These tasks are daunting, and many healthy adolescent girls have trouble accomplishing them easily. How much harder might it be for adolescent girls with a chronic health condition, such as HAIR-AN syndrome, to smoothly navigate the waters of adolescent turmoil to reach healthy adulthood, especially as it pertains to mental health?

HAIR-AN — a syndrome of hyperandrogenism (HA), insulin resistance (IR), and acanthosis nigricans (AN) — has been known for 30 years[21,22,23,24] and is a specific subtype of polycystic ovary syndrome (PCOS) characterized by the presence of severe insulin resistance[23,25]. PCOS is a disease of infrequent or absent ovulation and hyperandrogenism and is estimated to affect anywhere between 5–10%[26] and up to 12%[27,28] of females of reproductive age. Of those females with hyperandrogenism, approximately 5–10% have HAIR-AN syndrome[22,29], and up to 40% of adolescent patients who present with irregular periods may have HAIR-AN syndrome[30,31].

While many females with HAIR-AN syndrome have symptoms in adolescence or earlier, diagnosis is often delayed until adulthood[32,33]. Clinical manifestations of HAIR-AN syndrome include obesity, acne, hirsutism (i.e., growth of pigmented hair on chest, back, and lower abdomen), and acanthosis nigricans (i.e., darkly pigmented, velvety areas of the skin, usually found on the back of the neck, axillae, and in other skin fold areas). These clinical symptoms usually appear around puberty[34]. Additionally, due to insulin resistance and obesity, an adolescent has a greater propensity toward diabetes mellitus[35], and since menstrual cycles are often affected, an adolescent's chances of problems with fertility may also be increased[36].

### PSYCHOLOGICAL IMPACT OF HAIR-AN SYNDROME ON ADOLESCENT GIRLS

While many studies of HAIR-AN syndrome and PCOS discuss long-term health consequences of the disorders[24,25,35,37], little has been written about the mental health or psychological consequences of HAIR-AN syndrome for the adolescent girl[20,30,38]. In terms of mental health consequences, Omar et al. found that 24% of his sample of adolescents with HAIR-AN also reported depression[30]. Trent et al.[38] looked at health-related quality of life (HRQOL) in adolescent girls with PCOS and found that girls with PCOS had lower, but nonsignificant ($p$ = 0.07) scores on the mental health subscale of the Child-Health Questionnaire—Child Self-Report Form (CHQ—CF87) than healthy controls[38]. In another study with adolescents with PCOS, Trent et al. found that middle to late adolescents (M age = 16.9) were significantly more likely to express worry about their fertility than healthy controls[36].

In a study of youth with chronic health conditions (i.e., asthma, arthritis, diabetes) other than HAIR-AN or PCOS, Petersen et al. looked at HRQOL in children 8–16 years old (M = 12) and found that girls were significantly more likely than boys to have a negative emotional reaction regarding their chronic condition and were less likely to be accepting of it[39]. Additionally, in terms of HRQOL self-report, girls were significantly less likely than boys to be accepting of their physical problems and were more negatively affected by it emotionally. There was also a significant difference regarding emotional response to the quality of life in that younger children (8–12) reported a better emotional response to their chronic health condition than older ones (13–16), suggesting that as a teen ages, he/she will have a more negative emotional reaction to a chronic health condition[39]. These authors suggest that the changes that occur during puberty have a greater influence on girls than on boys. Additionally, it has been found that adolescent girls tend to report a lower HRQOL, especially with respect to physical self-image[40].

While the studies outlined above provide some data regarding the psychological impact of HAIR-AN and other chronic health conditions on adolescents, most studies regarding HAIR-AN do not assess psychological impact. However, some authors suggest that HAIR-AN or PCOS syndromes might be psychologically distressing[20,37]; these authors use words such as depressed, anxious, embarrassed, low self-image, and low self-esteem to delineate potential feelings an adolescent might experience when diagnosed with a chronic health condition such as HAIR-AN syndrome.

### Early Adolescence and Psychological Sequelae of HAIR-AN Syndrome

Early adolescence may be the most difficult stage of adolescence to navigate because it is the foundation on which the rest of adolescence is built and as such, will help determine an adolescent's ability to successfully complete middle and late adolescent tasks to emerge as a psychologically healthy adult. Early adolescence has significant physical changes, and as these changes occur, the girl with HAIR-AN syndrome will likely be faced with one or more unexpected consequences of her physical maturation, including acne, obesity, hirsutism, and/or nigrican acanthosis. The majority of girls diagnosed with HAIR-AN during this period will not have had to deal with chronic health conditions prior to the diagnosis and will likely be ill-prepared to do so, as will be their parents. The early adolescent girl may or may not embrace her physical maturation with joy, but whatever her attitude regarding the advancement toward womanhood, she likely did not expect complicating factors impacting her social and emotional worlds.

Additionally, early adolescents' cognitive capacities may not allow for a true awareness of the condition and its long-term effects. Early adolescents are just beginning to think abstractly[4,6] and they may have little true understanding of their condition. In fact, Trent et al. asked questions about PCOS and its effect on adolescent subjects' lives. Of those responding, 32% reported they did not understand the disorder, 48% understood somewhat, and 20% understood the disease well[36]. These data appear to suggest that while early adolescents begin to be able to think abstractly at times, these capacities fluctuate frequently, impacting their ability to fully appreciate the reality of their lives.

The social world of the early adolescent is filled with peers wherein the adolescent seeks validation, affiliation, and may seek close friendships, especially with female peers. The female adolescent is also beginning to explore her sexual self and may have some desire for dating or romantic relationships. The potential clinical symptoms associated with HAIR-AN syndrome may impede the young adolescent's ability to begin to form peer relationships of a stable, mature nature. Acne, obesity, hirsutism, and/or nigricans acanthosis may cause the young teen to be embarrassed, depressed, have low self-esteem, and a poor body image. Obesity, in particular, is looked on askance by the adolescent community. This increases the probability for eating disorders, especially when the age of onset for 85% of all eating disorders is between 11 and 20 years[41], and obese youth are rarely looked on kindly[9], predisposing them to unhealthy dieting practices and attempts to lose weight. Such practices may be particularly harmful in the young woman with HAIR-AN syndrome since the insulin resistance associated with it increases the likelihood of diabetes, and diet should be tailored to the disorder.

Substantial acne, dark pigmented skin, and excess hairiness may also wreak havoc with the young adolescent's sense of self in the social world. Additionally, these clinical manifestations of the disorder will also likely have impact in the young adolescent's emotional well-being and development. A sense of personal identity is a major developmental task of adolescence, and it begins in early adolescence with a preoccupation with the body and its changes, the trying on of different roles, and self-evaluation based on the acceptance or rejection of peers. A young adolescent's general psychological health might also be impacted, given that early adolescence is a time where teens often have wide fluctuations in mood. Moodiness, including anger, depression, anxiety, and withdrawal, may be intensified in the young teen with HAIR-AN syndrome.

An adolescent's ability to deal with her chronic illness will also likely be impacted by her relationship with her parents and their response to the diagnosis. Parental response will likely be mediated by parenting style. Child development researchers have extensively studied four parenting styles: (1) authoritative, (2) authoritarian, (3) indulgent, and (4) neglectful[42]. Authoritative parenting is characterized by high acceptance/involvement and moderately high strictness/supervision, and includes parental warmth, consistency, and nonpunitive punishment. Authoritarian parenting is characterized by low acceptance/involvement and very high strictness/supervision. Indulgent parenting has high acceptance/involvement and low strictness/supervision, and neglectful parenting is low on both dimensions. In general, adolescents with authoritative parents score highest on measures of psychological adjustment and those with authoritarian parents score lowest, with the other two parenting styles producing a mixed bag of results with regard to adjustment[43]. Thus, a youngster's response to her diagnosis will be somewhat dependent on parental response.

Authoritative parents will likely provide a level of support and firmness that the young adolescent needs, and development is likely to proceed as predicted. Authoritarian, indulgent, or neglectful parents will likely have a harder time providing appropriate, supportive parenting to their child with this chronic health condition. Regardless, parental response is very important in helping the adolescent to negotiate development with the chronic health condition and that response may be helpful or harmful.

#### Middle and Late Adolescence and Psychological Sequelae of HAIR-AN Syndrome

If a young adolescent navigates early adolescence fairly successfully and comes to terms with her chronic health condition, it would be expected that middle and late adolescence would proceed as expected. However, for those young adolescents who are unable to negotiate appropriate and fulfilling social relationships and emotional development, middle and late adolescence are likely to be problematic and may impede the girl's successful transition to adulthood in some domains. As noted earlier, physical maturity does not predict cognitive, social, and/or emotional maturity, and only as one successfully manages her chronic condition and moves forward is there a chance for full psychological adjustment.

Late adolescence may also carry with it additional concerns. By this time, a youth should have finished physical and cognitive development, allowing abstract thought. This will allow a late adolescent to view her condition in a context of time, and she may also be more aware at this point of the potential ramifications her condition has for future fertility[36]. Late adolescence is also a time when the young adult is attempting to consolidate relationships and a mature sexual identity. Concerns about sexual desirability and fertility may well come into play at this point, if they have not done so earlier, and as has been found, as a teen ages, she is less likely to have a positive emotional response to the chronic health condition[39], thus increasing the probability for depression and other psychological concerns to surface.

### CONCLUSIONS AND FUTURE DIRECTIONS

HAIR-AN syndrome is a disorder that affects a significant percentage of females and has clinical manifestations that will likely impact the psychosocial world of the developing adolescent as well as the physical one. From a clinical perspective, as well as from suggestions from the limited research available, it appears that the psychological sequelae of a diagnosis of HAIR-AN syndrome for an adolescent girl should be considered and addressed in working with the adolescent[20,30,36].

Adolescent medicine clinics that provide a full range of services, including mental health services, will most likely offer the best solution for the adolescent with HAIR-AN syndrome and her family. Often, a family will not or cannot seek mental health treatment apart from the medical environment in which they seek treatment for their child, so any intervention received will occur in the primary care or pediatric medical environment. If the medical environment can provide a full range of services, effective intervention can be provided.

Education regarding the syndrome and its potential effects is important, especially given an adolescent's concern with physical attractiveness. Various modalities of counseling are important adjuncts to medical treatment. Individual as well as group counseling should be considered. Group counseling can be especially therapeutic when a teen is placed in a supportive group with other young women with similar issues. This is important because youngsters often think no one else has the same problem as they do. The support they can obtain from others with similar issues can be immeasurably important in their continued successful progression toward adulthood. Family counseling may also be important, especially in families where parenting styles do not predict positive adjustment for the teen (i.e., authoritarian, indulgent, neglectful).

Research concerning HAIR-AN syndrome and PCOS should continue to look at aspects of quality of life for adolescents with these conditions, and it should also incorporate additional measures of psychological distress in order to obtain a better picture of how the syndrome affects girls psychologically. Long-term medical outcomes will be more positive if we can also have greater impact on the psychological impact of the disorder. Finally, education regarding the impact of chronic medical conditions on adolescents' mental and psychological well-being must be more fully incorporated into training for medical providers, especially those training to practice in primary care and pediatric settings. Early detection of potential psychological sequelae of medical conditions like HAIR-AN syndrome can lead to early intervention, and referral, if needed, thereby decreasing the probability of long-lasting negative impact of the disorder on psychological function.
